# Evaluation of three canine γ-crystallins (*CRYGB*, *CRYGC*, and *CRYGS*) as candidates for hereditary cataracts in the dachshund

**Published:** 2007-01-31

**Authors:** Christina Müller, Anne Wöhlke, Ottmar Distl

**Affiliations:** Institute for Animal Breeding and Genetics, University of Veterinary Medicine Hannover, Foundation, Hannover, Germany

## Abstract

**Purpose:**

We analyzed the γ-crystallin genes *CRYGB*, *CRYGC*, and *CRYGS* in the dog and tested single nucleotide polymorphisms (SNPs) for linkage and association with primary noncongenital cataract (CAT) in the dachshund, a popular dog breed. The crystallin genes may be involved in the pathogenesis of canine CAT as shown in humans and mice.

**Methods:**

We sequenced all exons and their flanking intronic regions of the *CRYGB*, *CRYGC*, and *CRYGS* genes and in addition, the complete cDNA of these three genes using lens tissue from CAT-affected and unaffected dogs of several breeds. After examining BLASTN analyses, we compared the gene structure with the predicted genes in the current dog genome assembly and the orthologs of humans and mice.

**Results:**

The search for SNPs within these crystallin genes revealed a total of five polymorphisms. As both CAT-affected and unaffected dogs shared identical haplotypes, there was no cosegregation of the SNP alleles with the affected animals. Expression did not differ among CAT-affected and unaffected dogs.

**Conclusions:**

The polymorphisms reported for *CRYGB*, *CRYGC*, and *CRYGS* can be excluded as causative mutations for the CAT phenotype in the wire- and smooth-haired dachshund. The canine cataract gene orthologs described here may serve as a valuable resource for further studies in other dog breeds to develop a canine model. Many different dog breeds are affected by CAT. The use of the SNPs presented in this paper can facilitate the screening of more dog breeds.

## Introduction

Primary hereditary cataracts are common in purebred dogs, affecting over 120 breeds. Cataracts frequently cause visual impairment and are a major cause of blindness in dogs [1-6]. Inheritance of noncongenital cataracts has been demonstrated in several dog breeds, e.g., the golden and labrador retrievers [7,8], German shepherd [9], West Highland white terrier [10], American cocker spaniel [11], Tibetan terrier [12], Afghan hound [13], standard poodle [14,15], and the Entlebucher mountain dog [16]. As the dachshund is a breed predisposed to primary noncongenital cataract (CAT), it is assumed that these cataracts are also hereditary [2,4].

Dachshunds are bred in three coat varieties (long-haired, smooth-haired, and wire-haired) and three different sizes (standard, dwarf, and rabbit). Dwarf- and rabbit-sized dachshunds are referred to in this investigation as miniature dachshunds. The prevalence of CAT in the long-haired dachshund in North America is 2.10% [6]. In Germany, the prevalence has been estimated to be 3.21% for the long-haired, 1.87% for the smooth-haired, and 4.80% for the wire-haired dachshund [17]. In an animal threshold model, the heritabilities for CAT were 0.39±0.06 (wire-haired), 0.08±011 (long-haired), and 0.72±0.28 (short-haired) [17].

The transparency and high refractive index of the eye lens is achieved by a regular arrangement of the lens fiber cells and by a high concentration and the supramolecular organization of the lens-specific proteins, the crystallins, within each fiber cell [18].

The crystallin proteins are the major structural components of the eye lens that constitute 80-90% of its soluble proteins. These proteins are divided into three classes, α-, β-, and γ-crystallins, which form two protein superfamilies: the α-crystallin superfamily and the β-/γ-crystallin superfamily [19,20]. The common characteristic of the β-/γ-superfamily is a unique folding structure, the Greek-key motif. Each of the β- and γ-crystallins has two domains, with each domain being composed of two extremely stable protein structures, the so-called Greek-key structural motifs. These structures allow a dense packing of proteins in the ocular lens [21,22]. Any structural alterations of these proteins can disturb the highly ordered tissue architecture and can lead to opacity. Because of that, the genes that encode these proteins are obvious candidate genes for cataracts.

The γ-crystallins are encoded by the *CRYG* genes. Six members of the *CRYG* family (*CRYGA*-*CRYGF*) are located in a cluster on mouse chromosome 1 [23-25] and on human chromosome 2, respectively [26-28]. The seventh *CRYG* gene, *CRYGS*, maps to mouse chromosome 16 and human chromosome 3 [29,30]. Also in the dog, the *CRYG* genes are located in a cluster on dog chromosome (CFA) 37 with the exception of *CRYGS*, which maps to CFA34 [31]. In mice, mutations identified in different *CRYG* genes are known to cause dominant or recessive cataracts [32-38]. Also in humans, several hereditary cataracts have been shown to be caused by mutations in the *CRYG* genes [39-43]. In this report, we provide the complete sequence and the genomic sequences of all exons of *CRYGB*, *CRYGC,* and *CRYGS.* In addition, we test single nucleotide polymorphisms for linkage and association in dachshunds and compare expression of mRNA in lenses of dogs affected by primary cataract and an unaffected control dog.

## Methods

### Animals, phenotypic data, and DNA specimens

Ophthalmological data for the dachshunds were provided by the Dortmunder Kreis (DOK), which is the German panel of the European Eye Scheme for diagnosis of inherited eye diseases in animals. The German Dachshund Club 1888 e.V. (DTK) supplied pedigree data and we identified pedigrees with multiple CAT-affected dogs. For the present analysis, we chose 24 dogs from four different dachshund families. Most of the animals (14 dogs) came from a standard-sized wire-haired family, whereas seven wire-haired miniature dachshunds were from two different families. The other three dogs were smooth-haired standard-sized dachshunds. Altogether, this study included 17 CAT-affected dachshunds. The signs of CAT in these dogs differed in regard to stage. In seven dogs an immature cataract was diagnosed, in eight dogs an incipient cataract, and in two dogs a mature cataract. Most of the affected dogs included in the analysis had an opacification localized in the cortex of the lens (82.4%). Lens opacity was additionally found in the capsule (one dog) and the nucleus (five dogs). In one dog only the nucleus was affected; two dogs showed only capsular opacifications. Both eyes were affected in ten animals, while alterations were found only in the lens of the left eye in the other seven. Most of the dogs (about 70%) were examined two to three times. CAT was first diagnosed at a mean age of 3.67±2.14 years. At least one unaffected dog was investigated from each family. The seven unaffected dogs were between 5.1 and 9.7 years old at the last ophthalmological examination.

We also tested four unaffected dogs from other breeds (dalmatian, German shepherd mix, Hanoverian hound, great dane) as control animals.

Two milliliters of heparinized blood were obtained from each dog, and DNA was extracted using QIAamp 96 DNA Blood kit (Qiagen, Hilden, Germany).

For cDNA analysis of the three genes, we used lens tissue of seven dogs of six different breeds (mixed breed, German shepherd dog, dachshund mix, Jack Russell terrier, Tibetan terrier, and Yorkshire terrier). Six of the seven dogs underwent cataract surgery; one dog (mixed breed) with normal lenses was used as reference. The cataract surgery was done using the phacoemulsification method with ultrasound. After removal from eye, the lens tissue was conserved using RNA-later solution (Qiagen). The RNA was extracted from dog lens tissue using the Nucleospin RNA II-Kit (Machery-Nagel, Düren, Germany) and transcribed into cDNA using SuperScript III Reverse Transcriptase (Invitrogen, Karlsruhe, Germany).

### Structural analysis of the canine *CRYGB*, *CRYGC*, and *CRYGS* gene

We searched the dog-expressed sequence tag (EST) archive for ESTs by cross-species BLAST searches with the corresponding human reference mRNA sequences for *CRYGB* (NM_005210), *CRYGC* (NM_020989), and *CRYGS* (NM_017541). We found a canine EST (DN866034) isolated from dog lens tissue with 88% identity to the human *CRYGB* mRNA sequence. A significant match to this canine EST was identified on canine chromosome 37 (NW_876304.1|Cfa37_WGA83_2) by means of BLASTN searches of this canine EST against the dog genome assembly (Dog genome assembly 2.1).

For *CRYGC* we found a canine EST (DN867687) with 87% identity to the human mRNA sequence. A significant match to this canine EST was identified on canine chromosome 37 (NW_876304.1|Cfa37_WGA83_2) [44].

We also found a canine EST (DN867380) isolated from beagle lens tissue with 90% identity to the human *CRYGS* mRNA sequence. A significant match to this canine EST was identified on canine chromosome 34 (NW_876301.1|Cfa34_WGA80_2) by means of BLASTN searches of this canine EST against the dog genome assembly (Dog genome assembly 2.1). The genomic structure of the canine *CRYGB*, *CRYGC*, and *CRYGS* genes were determined with the Spidey mRNA-to-genomic alignment program. We verified the canine ESTs by sequencing the cDNA of all three genes isolated from the lens of seven dogs. The primers were designed in such a way that the open reading frames of the three genes were amplified. All reverse primers were located downstream from the stop codons of the three genes. The forward primers included the start codon (*CRYGB* and *CRYGS*) or were located a few bases upstream of the start codon (*CRYGC*; Table 1).

**Table 1:** γ-Crystallin PCR primers.

**Gene**	**Target**	**Primer**	**Sequence (5'-3') of primers**	**Annealing temperature (°C)**	**Product size (bp)**
CRYGB	Exon 1 and Exon 2	CRYGB_Ex1_Ex2_F	TGGTTTAATTGCCTTTGAGG	58	929
		CRYGB_Ex1_Ex2_R	AAGCAAGCACCACAGAGTTC		
	Exon 3	CRYGB_Ex3_F	TTGGAAGCAAACCTAGACTCC	58	577
		CRYGB_Ex3_R	TCCCCCTTAGAAGACAGTATTTC		
CRYGC	Exon 1 and 5' UTR	CRYGC_Ex1_F	CACTAAGAATCCAAATAAAAGCAAC	58	390
		CRYGC_Ex1_R	CGTAGAAGGTGATCTGCAAAG		
	Exon2	CRYGC_Ex2_F	AAGGTGAGCGGGATACAAG	58	494
		CRYGC_Ex2_R	CTGGCTTTGTGCATTTGTC		
	Exon 3 and 3' UTR	CRYGC_Ex3_F	ACACACAGCCATCTCAGAGTC	58	577
		CRYGC_Ex3_R	CATTTCACTTTGCAGAGCTTC		
CRYGS	Exon 1 and 5' UTR	CRYGS_Ex1_F	TCAATAGCCTCTAAATGACTGACTC	58	290
		CRYGS_Ex1_R	GTACATTGGAAAAGAGGAAACG		
	Exon2	CRYGS_Ex2_F	GCCAGAGGATAGGTGTTGTG	58	495
		CRYGS_Ex2_R	GGGAGGGAGTAGGGAAAAG		
	Exon 3 and 3' UTR	CRYGS_Ex3_F	CATGCTGTTCTCGGAGTTG	58	468
		CRYGS_Ex3_R	AGGCATTACAGTCAACACTGG		

CRYGB	cDNA CRYGB*	CRYGB_F	CATGGGAAAGATCACCTTCTAC	58	567
		CRYGB_R	TTGGATTCTAAAGGACAAAAGTG		
CRYGC	cDNA CRYGC**	CRYGC_F	GCCAGTCGCACTGAACTC	58	603
		CRYGC_R	TTAGGTTCCAAACTGAGAAAATG		
CRYGS	cDNA CRYGS^#^	CRYGS_F	ACCAATCTATGCAACAAAATGTC	58	645
		CRYGS_R	GCCAATTGTTTTATTTATGATGC		

Shown are the PCR primers for the amplification of genomic canine *CRYGB*, *CRYGC*, and *CRYGS* exons with their flanking intronic regions and PCR primers for the amplification of the cDNA of the canine *CRYGB*, *CRYGC*, and *CRYGS* genes. The asterisk indicates that the forward primer is located 1 bp upstream of the start codon and the reverse primer is located 38 bp downstream of the stop codon. The double asterisk indicates that the forward primer is located 39 bp upstream of the start codon and the reverse primer is located 39 bp downstream of the stop codon. The sharp (hash mark) indicates that the forward primer is located 8 bp upstream of the start codon and the reverse primer is located 90 bp downstream of the stop codon.

### Mutation analysis

For evaluation of *CRYGB*, *CRYGC*, and *CRYGS* as candidate genes for CAT in the dachshund, we sequenced all exons and their flanking intronic regions of the three genes for the animals mentioned above (Table 1). All PCRs were performed in 50 μl reactions using 50 pmol of each primer, 100 μM dNTPs, 2 U *Taq*-DNA-Polymerase (Q-BIOgene, Heidelberg, Germany) in the reaction buffer supplied by the manufacturer, and 10X PCR Enhancer (Invitrogen, Karlsruhe, Germany) for 2 μl template DNA or cDNA, respectively. The PCR conditions were as follows: 95 °C for 4 min followed by 34 cycles of 94 °C for 30 s, annealing temperature of 58 °C for 45 s, 72 °C for 45 s, and 4 °C for 10 min. All PCR products were cleaned using the Nucleo-Fast PCR purification kit (Machery-Nagel) and directly sequenced with the DYEnamic ET Terminator kit (Amersham Biosciences, Freiburg, Germany) and a MegaBACE 1000-capillary sequencer (Amersham Biosciences). PCR primers were generated with the Primer3 program based on the canine ESTs (DN866034, DN867687, and DN867380) and the canine genomic sequences (NW_876304.1|Cfa37_WGA83_2, NW_876304.1|Cfa37_WGA83_2, and NW_876301.1|Cfa34_WGA80_2). Sequence data were analyzed with Sequencher 4.7 (GeneCodes, Ann Arbor, MI).

### Nonparametric linkage and association analyses

A nonparametric multipoint linkage analysis was employed for the four dachshund families. This analysis was based on allele sharing by identical-by-descent methods and the MERLIN 1.0.1 software [45]. Haplotypes were estimated using MERLIN 1.0.1 using the option "best". A case-control analysis based on χ^2^ tests for genotypes, alleles, and trends of the most prevalent allele was also performed for the dachshund families. The CASECONTROL and ALLELE procedures of SAS were used for association tests, tests for Hardy-Weinberg equilibrium of genotype frequencies, and the estimation of allele frequencies.

## Results & Discussion

### Position of *CRYGB*, *CRYGC*, and *CRYGS* on canine chromosomes

The canine ESTs for *CRYGB* (DN866034) and *CRYGS* (DN867380), which were found by cross-species BLAST searches with the corresponding human reference mRNA sequences, mapped to the same positions as the annotated canine genes for *CRYGB* (LOC488497) and *CRYGS* (LOC607506; Table 2). For *CRYGC*, no annotated canine gene is available but the *CRYGC* EST (DN867687) mapped between canine *CRYGB* (LOC488497) and *CRYGD* (LOC488495). This assumed position of *CRYGC* in dogs agrees with all other investigated species [23-25]. A recent study tried to obtain sequence tagged sites (STS) for *CRYGB* and other candidate genes for primary cataracts in the dog [31]. The putative canine *CRYGB* amplicon was located at 20,124,535-20,124,930 on CFA37 in this previous study. This location is close to LOC609894, similar to *CRYGE*. However, the canine *CRYGB* gene (LOC488497) as verified in our study is located on CFA37 at 19.440-19.445 Mb. Comparisons between the primer sequence of the *CRYGB* amplicon (AACN010184836) used by Hunter et al. [31] and the predicted canine mRNA sequences for *CRYGB* (XM_545618.2) and *CRYGE* (XM_847242.1) showed that the canine mRNA sequence for *CRYGB* (XM_545618.2) had less homology (95%) to the sequence under accession number AACN010184836 than the canine mRNA sequence for *CRYGE* (97%). Homology between the predicted canine mRNA sequences for *CRYGB* (XM_545618.2) and the human *CRYGB* mRNA sequence (NM_005210) was 88%, but only 81% between predicted canine *CRYGE* mRNA (XM_847242.1) and human *CRYGB* mRNA (NM_005210). The canine EST (DN86603) we used to determine the genomic structure of the canine *CRYGB* gene mapped to the same position as the annotated canine locus for *CRYGB* (LOC488497). The *CRYGB* cDNA sequences of all seven dogs analyzed in the present study perfectly matched the sequence of the canine *CRYGB* EST.

**Table 2:** SNP analysis.

**Position of gene (Mb)**	**SNP**	**HET (%)**	**PIC (%)**	**Z-mean**	**P Z-mean**	**LOD Score**	**P LOD Score**	**χ^2^ allele**	**P allele**	**χ^2^ genotype**	**P genotype**
*CRYGB* on CFA37 19.440-19.442											
	*LOC488497:g.2537C>A*	45.8	31.7	0.32	0.4	0.16	0.2	0.02	0.88	3.17	0.20
	*LOC488497:g.4348T>C*	26.1	24.6	0.32	0.4	0.16	0.2	0.23	0.63	2.83	0.24
*CRYGC* on CFA37 19.431-19.433											
	*DN867687:c.364C>T*	16.7	19.5	0.32	0.4	0.16	0.2	0.52	0.47	0.5	0.78
	*DN867687:c.379C>T*	25	23.9	0.32	0.4	0.16	0.2	0.32	0.57	2.88	0.24
*CRYGS* on CFA34 22.166-22.172											
	DN867380:c.*7G>A	33.3	30.5	0.22	0.4	0.15	0.2	0.13	0.71	2.91	0.23

Shown are heterozygosity (HET), polymorphism information content (PIC), chromosome-wide multipoint test statistics Z-mean and LOD Score, their error probabilities (p Z-mean, p LOD Score), χ^2^-tests for allele and genotype distribution of the case-control analysis and their corresponding error probabilities (P) for the SNPs in the dachshund families. According to the SNP nomenclature, the asterik indicates the position of a changed nucleotide 3' of the translation stop codon.

The crystallin genes are similar genes that show high sequence homologies to each other. We assume that the problems of the previous study to place the putative *CRYGB* amplicon to the predicted canine *CRYGB* location was due to assortment of the wrong sequence (AACN010184836) for primer design of the *CRYGB* amplicon.

### Genomic organization of canine *CRYGB*

**Figure 1:**
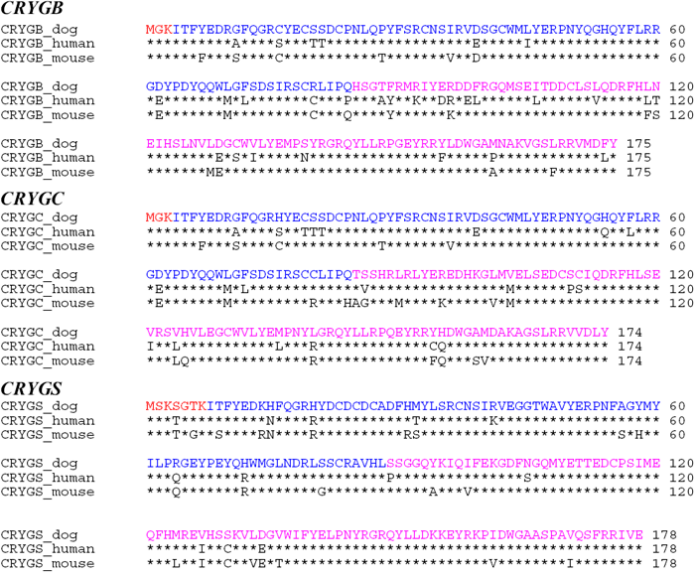
γ-Crystallin protein alignment. Shown are the alignment of the canine CRYGB protein (175 amino acids), the canine CRYGC protein (174 amino acids), and the canine CRYGS protein (178 amino acids) derived from our sequenced cDNA with the known orthologous protein sequences. The sequences were derived from GenBank entries with the following accession numbers: NP_005201 (human CRYGB), NP_658906 (mouse CRYGB), NP_066269 (human CRYGC), NP_031801 (mouse CRYGC), NP_060011 (human CRYGS) and NP_034097 (mouse CRYGS). Residues identical to the dog are indicated by asterisks. The three exons are labeled by different colors. All exons included only complete triplets.

The canine *CRYGB* gene was found to contain all three exons and two introns that are present in the orthologous human gene, and the canine exon/intron boundaries conformed perfectly to the GT/AG rule. The sizes of all three exons of the canine *CRYGB* gene were identical to those of the human *CRYGB* gene. Analysis of the 648 bp of the canine *CRYGB* EST (DN866034) revealed an open reading frame of 528 bp predicting a protein of 175 amino acids. The canine CRYGB protein displayed 84.6% similarity to the human CRYGB protein and 91.4% similarity to the mouse CRYGB protein (Figure 1).

The canine EST contained 29 nucleotides before the start codon in exon 1, and 91 nucleotides after the stop codon in exon 3. The polyadenylation signal AAUAAA was located 37 bp downstream of the stop codon.

The cDNA sequences of lens tissue of the seven dogs perfectly matched the sequences of the canine ESTs. Only the 5' and the 3' ends were shorter due to primer position.

The gene structure described here is in contrast to the annotated structure of the canine *CRYGB* gene (LOC488497), which was derived by automated computational analysis (Dog genome assembly 2.1). Under this accession number, the canine *CRYGB* gene has five exons, with exon 3 corresponding to human exon 1, exon 4 corresponding to human exon 2, and exon 5 corresponding to human exon 3. The canine EST and our sequenced *CRYGB* cDNA contained only the predicted canine exons 3, 4, and 5. No canine EST was found for the predicted exons 1 and 2.

The γ-crystallin genes are considered to be highly conserved genes, which are similar among the different species. In all mammals examined to date, the γ-crystallin genes have a three-exon-structure: a short first exon, which encodes the start codon, and the short NH_2_-terminal "arm". The other two exons encode the two structural domains, each of which contains two Greek key motifs [21,22,46]. We could not verify the predicted canine exons 1 and 2 as described in LOC488497. Due to the fact that the gene structure of LOC488497 was derived by automated computational analysis, it is possible that the predicted exons 1 and 2 do not exist.

### Genomic organization of canine *CRYGC*

The canine *CRYGC* gene contained all three exons and two introns that are present in the orthologous human gene. The sizes of all three exons of the canine *CRYGC* gene were identical to the human *CRYGC* gene. The analysis of the 669 bp of the canine *CRYGC* EST (DN867687) revealed an open reading frame of 525 bp, predicting a protein of 174 amino acids. As the canine EST contained 89 nucleotides before the start codon in exon 1 and 55 nucleotides after the stop codon in exon 3, we assumed that the 5'- and 3'-UTR of the canine *CRYGC* were included [44]. The canine CRYGC protein displayed 87.4% similarity to the mouse CRYGC and 87.9% similarity to the human CRYGC protein (Figure 1).

### Genomic organization of canine *CRYGS*

The canine *CRYGS* gene also had three exons interrupted by two introns as does the orthologous human gene. The canine exon/intron boundaries conformed perfectly to the GT/AG rule, and the sizes of all three exons of the canine *CRYGS* gene were identical to those of the human *CRYGS* gene. Analysis of the 680 bp of the canine *CRYGS* EST (DN867380) revealed an open reading frame of 537 bp, predicting a protein of 178 amino acids. The canine CRYGS protein displayed 93.3% similarity to the human CRYGS protein and 87.1% similarity to the mouse CRYGS protein (Figure 1). The canine EST contained 39 nucleotides before the start codon in exon 1 and 104 nucleotides after the stop codon in exon 3. The polyadenylation signal AAUAAA was located 76 bp downstream of the stop codon.

 LOC607506 lists seven isoforms of the canine *CRYGS* gene which were derived by automated computational analysis (Dog genome assembly 2.1). The isoform with the transcript ID XM_844792.1 agrees with the results of our study. The other isoforms have additional exons, which were not confirmed in our analysis, or have different exon sizes that do not fit into the canine EST.

**Figure 2:**
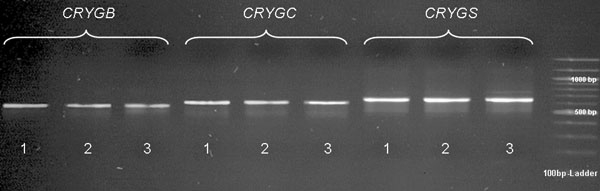
γ-Crystallin cDNA analysis. Bands of cDNA PCR products of the lens tissue of two dogs affected by CAT and an unaffected dog for each gene (*CRYGB*, 567 bp; *CRYGC*, 603 bp; and *CRYGS*, 645 bp) on an agarose gel. In the gel, band 1=mixed breed, unaffected; band 2=dachshund mix, affected; band 3=German shepherd, affected. The cDNAs of the other four dogs did not differ in sequence and product size.

To confirm the results of the structure analyses of the three genes, it would be necessary to produce a full-length cDNA using RACE methods. For this purpose it is necessary to obtain complete RNA with intact 3' and 5' ends. It was possible to gain the cDNA sequence of all three genes without the 5' ends. The cDNA sequences of all dogs perfectly matched to the sequences of the canine ESTs. Our sequenced cDNA of the three genes contained all exons corresponding to the ESTs described (DN866034, DN867687, and DN867380) and a few bases upstream from the start codons and downstream from the stop codons, respectively. Figure 2 shows the cDNAs from the lens of two CAT-affected dogs and an unaffected dog for each gene on an agarose gel. The cDNAs of the other four dogs did not differ in sequence and product size. However, even after several attempts, it was not possible to receive full-length cDNAs of the investigated genes.

### Polymorphisms within the canine *CRYGB*, *CRYGC*, and *CRYGS* gene

The search for sequence variations within the three genes revealed a total of five SNPs as shown in Table 3. Of these five SNPs, two were located in the exon sequence of *CRYGC* while another was located in the exon sequence of *CRYGB*. The exonic SNP of *CRYGB* was a T>C transition in exon 3, which changes a GTT triplet to a GTC triplet. Both triplets code for valine and thus do not alter the amino acid sequence of *CRYGB*. In the *CRYGC* gene, a C/T transition at position 112 of exon 3 was observed only in the wire-haired dachshunds. This transition changes a CGC triplet into a TGC triplet and thus causes an amino acid change from arginine (R) to cysteine (C). This means a change from a charged alkaline amino acid to a neutral amino acid with a nonpolar side chain. Multi species protein sequence comparisons between human (R; accession number NP_066269), mice (R; accession number NP_031801), rat (R; accession number XP_343583), and cattle (R; accession number NP_001013613) showed that this position was not variable between the known orthologous *CRYGC* proteins.

**Table 3:** Nucleotide polymorphisms within the canine *CRYGB*, *CRYGC*, and *CRYGS* genes.

**Gene**	**Location of polymorphic site**	**Position and nucleotide polymorphism**	**Allele frequencies**	**Genotype frequencies**
*CRYGB*	Intron 1	NC_006619.2:g.2537C>A	0.71	50/43/7
	Exon 3	NC_006619.2:g.4348T>C	0.80	67/26/7
*CRYGC*	Exon 3^#^	DN867687:c.364C>T	0.89	82/14/4
	Exon 3	DN867687:c.379C>T	0.86	75/21/4
*CRYGS*	3'UTR	DN867380:c.*7G>A	0.75	61/29/11

Shown are the locations of nucleotide polymorphisms within the canine *CRYGB*, *CRYGC*, and *CRYGS* genes, their positions in the canine sequence used for the respective gene and SNP allele frequencies for the wild-type allele and distribution of SNP genotypes as percent of animals (homozygous for allele 1, and heterozygous and homozygous for allele 2). According to the SNP nomenclature, the asterik indicates the position of a changed nucleotide 3' of the translation stop codon. The sharp (hash mark) indicates that this SNP showed only a polymorphism in the wire-haired dachshunds.

The second exonic SNP of *CRYGC* was found at position 127 of exon 3. This C/T SNP changes a CTG triplet to a TTG triplet, which has no effect on the amino acid sequence of *CRYGC*. Except for DN867687:c.364C>T, all other SNPs were polymorphic in all six examined breeds (Table 3). None of the polymorphisms affected the splice sites in the investigated genes.

### Linkage and association analyses for *CRYGB*, *CRYGC*, and *CRYGS*

Table 2 shows the results of the nonparametric multipoint linkage analysis in the dachshunds. All SNP alleles were in Hardy-Weinberg equilibrium. The highest Z-mean value was 0.32 and the highest LOD score was 0.16, while the error probabilities ranged from 0.2 to 0.4. The maximum achievable Z-mean was 29.85 and the corresponding value for the LOD score was 5.52. These values indicated that the pedigrees used had enough power to detect significant linkage. There were also no significant results from the case-control χ^2^-tests for the dachshund families. The χ^2^ test statistics for allelic distributions between cases and controls ranged from 0.02 to 0.52 and their error probabilities from 0.88 to 0.47. Similar results were obtained for the distributions of genotypes between cases and controls (χ^2^ from 0.50 to 3.17 with error probabilities from 0.78 to 0.20).

Therefore, it is unlikely that the *CRYGB*, *CRYGC*, and *CRYGS* genes are involved in the pathogenesis of CAT in these wire- and smooth-haired dachshunds.

None of the five polymorphisms identified in this study proved to be a causal mutation for CAT in canine *CRYGB*, *CRYGC*, and *CRYGS* exons and exon/intron junctions in the CAT-affected wire- and smooth-haired dachshunds from our pedigrees. In addition, expression analysis of these genes in six affected dogs and a control dog did not reveal any differences in the bands on an agarose gel. So it seems unlikely that a mutation outside of the genomic regions analyzed here possibly affects *CRYGB*, *CRYGC* or *CRYGS* expression. However, the SNPs identified here may be useful to test *CRYGB*, *CRYGC*, and *CRYGS* as candidate genes in other dog breeds.
